# New Cytogenetic Data for the Neottieae Tribe (Orchidaceae) in the Mediterranean Region

**DOI:** 10.3390/plants13131776

**Published:** 2024-06-27

**Authors:** Alessio Turco, Robert Philipp Wagensommer, Antonella Albano, Pietro Medagli, Saverio D’Emerico

**Affiliations:** 1 Faculty of Education, Free University of Bozen-Bolzano, 39042 Brixen-Bressanone, Italy; alessio.turco@unibz.it; 2 Department of Biological and Environmental Sciences and Technologies, University of the Salento, 73100 Lecce, Italy; antonella.albano@unisalento.it (A.A.); pietro.medagli@unisalento.it (P.M.); 3 University of Bari “Aldo Moro”, 70125 Bari, Italy; sdeme@yahoo.it

**Keywords:** *Cephalanthera*, chromosome alterations, *Epipactis*, karyosystematic, *Limodorum*, *Neottia*

## Abstract

This work presents a summary of cytogenetic data, including new information, on several species within the tribe Neottieae, with an update of the karyotype for 23 species belonging to the genera *Cephalanthera*, *Limodorum*, *Epipactis*, and *Neottia* (including *Listera*). Each of these four genera also presents distinctive chromosomal features, such as bimodal karyotypes. Our research includes insights into the distribution of constitutive heterochromatin, measured using C-banding and, in some cases, specific fluorochromes for the detection of A-T- and G-C-rich DNA. In the *Epipactis* group, it is noteworthy that when using the Giemsa banding technique, certain species (e.g., *E. placentina*, *E. meridionalis*) with a chromosome number of 2n = 38 were observed to exhibit a conspicuous wide band of constitutive heterochromatin on the long arm of the third pair in a subcentromeric position, resembling what has been observed in *E. helleborine*. These differences also have the potential to contribute to the diversification of these species. Based on the karyological results obtained, a hypothesis regarding the origin of certain species within the *E. helleborine* group is proposed. Additionally, karyological analyses conducted on a specimen of *E. microphylla* revealed chromosome counts ranging from 36 to 40. Somatic metaphases exhibited evident structural alterations in certain chromosomes, showing rearrangements probably caused by translocation phenomena. Based on the data obtained from the species within the studied genera, it is conceivable that variations in chromosomes, both structurally and in the distribution of constitutive heterochromatin, exert a significant influence on the evolution of the karyotype. Moreover, in many entities belonging to the Neottieae tribe, these processes may also contribute to the diversification of the phenotype in some instances.

## 1. Introduction

According to Quentin [1] and the Plants of the World Online database [2], the Neottieae tribe occurs in the Mediterranean region and includes four genera: *Cephalanthera* Rich., *Limodorum* Boehm., *Epipactis* Zinn, and *Neottia* Guett., which includes the former genus *Listera* R.Br.

This tribe is characterised by autogamous, cleistogamous, and saprophytic species, and is further distinguished by unique morphological features, ecological adaptations, and evolutionary significance [3,4]. Furthermore, these orchids also play vital roles in their ecosystems by forming symbiotic relationships with mycorrhizal fungi [5].

In Europe, Delforge [6] reported about 80 Neottieae species, including varieties, grouped into the genera *Cephalanthera* (9), *Epipactis* (67), *Limodorum* (2), and *Neottia* (3). However, eight years later, the tally had only increased noticeably for *Epipactis*, reflecting the recognition of many local endemics by European orchid taxonomists [7]. In contrast, POWO [2] reports about 44 species. On the other hand, despite the increasing discovery of new species in the *Epipactis* genus, no further cytogenetic analyses have been conducted, although there have been numerous morphological, distributional, ecological, conservational, biochemical, and molecular analyses [5,8,9,10,11,12,13,14,15]. 

Previous studies of the chromosomes of Neottieae species have revealed a wide range of chromosome counts and configurations across genera and species [3,4,16,17,18,19,20,21,22,23,24,25,26,27,28]. These variations in chromosome number, often linked to karyotype diversity, reflect the dynamic evolutionary processes shaping the genetic makeup of these plants [29]. 

In this tribe, all species are characterised by a bimodal karyotype, consisting of a few long chromosomes and numerous small ones [17,18,19,20,24,25,26,28,30]. Bimodal chromosomes are characterised by changes in their structure or composition resulting from various genetic mechanisms [31,32,33,34,35,36,37,38,39]. Common rearrangements in plant bimodal chromosomes include fusion, fission, chromosomal translocation, inversions, and deletions [31,40,41,42,43]. Structural abnormalities such as rings or isochromosomes can also arise [44,45]. Methods such as genome sequencing, cytogenetic analysis, and gene editing allow researchers to investigate the genetic basis of these alterations [37,46,47].

Chromosomal data can provide clues to the mechanisms driving speciation, hybridisation, and genetic diversity within and among species [48]. Exploring the chromosomal evolution of Neottieae can, thus, shed light on their adaptation to diverse habitats and ecological niches. The study of chromosomes in Neottieae contributes to a broader understanding of orchid evolution and biodiversity [4,11]. 

In this study, we revisited existing cytogenetic data on representatives of the tribe Neottieae, integrating earlier findings with later findings to interpret chromosome evolution. Furthermore, we considered cases of variation in genotype and phenotype in some species, attributable to both structural rearrangements of chromosomes and differences in the distribution of constitutive heterochromatin.

Regarding the techniques used, our work suggests that Feulgen staining and Giemsa C-banding remain effective methods, complementary to molecular cytogenetics, for studying heteromorphic variations and characterising marker chromosomes or other structural rearrangements involving chromosomes.

## 2. Results

**Figure 1:**
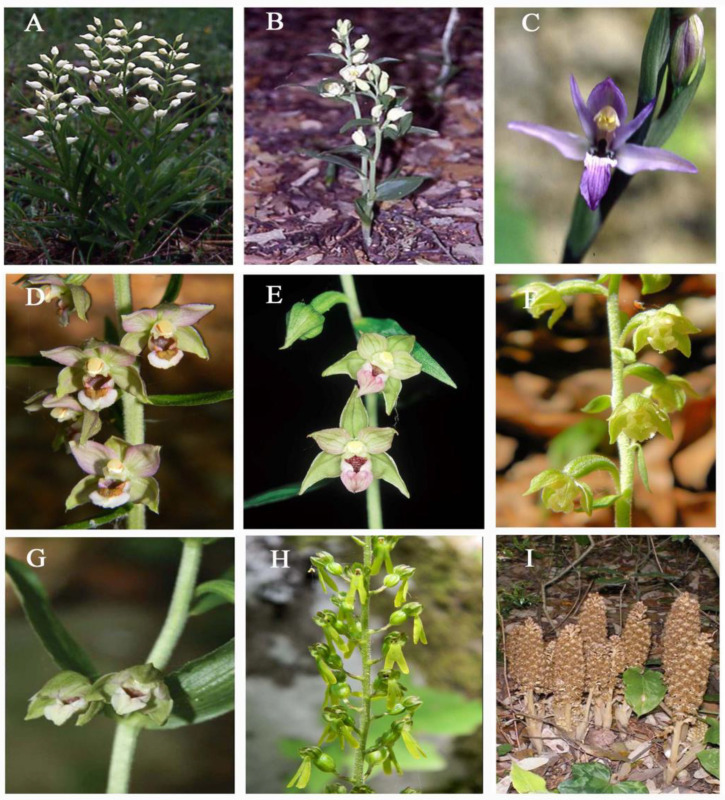
Representative species of *Cephalanthera* Rich., *Limodorum* Boehm., *Epipactis* Zinn, and *Neottia* Guett. discussed in the present study. (**A**) *Cephalanthera longifolia* (L.) Fritsch; (**B**) *C. damasonium* (Mill.) Druce; (**C**) *Limodorum abortivum* (L.) Sw.; (**D**) *Epipactis helleborine* (L.) Crantz; (**E**) *E. meridionalis* H. Baumann & R. Lorenz; (**F**) *E. microphylla* (Ehrh.) Sw.; (**G**) *E. cupaniana* C. Brullo, D’Emerico & Pulv.; (**H**) *Neottia ovata* (L.) Hartm.; (**I**) *N. nidus-avis* (L.) Rich.

Representative species from the *Cephalanthera*, *Limodorum*, *Epipactis*, and *Neottia* genera are depicted in Figure 1. Some of these species yielded novel karyological information, while for others, existing data were revised using IdeoKar 1.2 software.

The analysed parameters associated with the species belonging to *Cephalanthera*, *Limodorum*, *Epipactis*, and *Neottia* (including *Listera*) are shown in Table 1.

**Figure 2:**
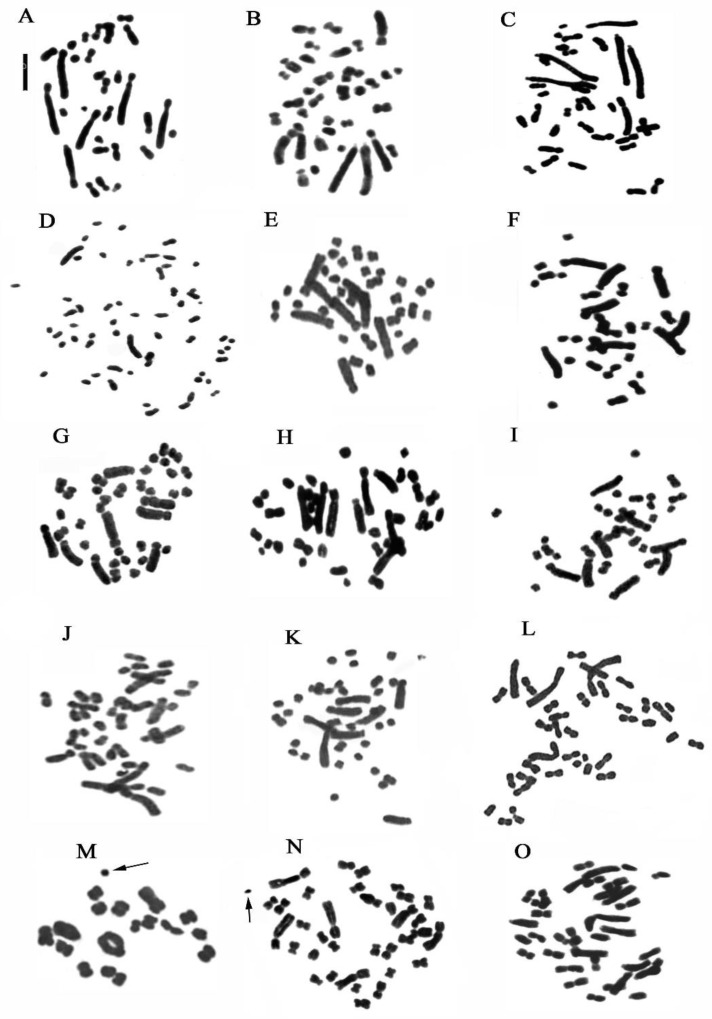
Metaphase chromosomes of (**A**) *Cephalanthera longifolia*, 2n = 32; (**B**) *C. damasonium* Sardinia, 2n = 36; (**C**) *C. damasonium* Continental Italy, 2n = 36; (**D**) *Limodorum abortivum*, 2n = 56; (**E**) *Epipactis helleborine*, 2n = 40; (**F**) *E. placentina*, 2n = 38; (**G**) *E. meridionalis*, 2n = 38; (**H**) *E. microphylla*, 2n = 40; (**I**) *E. cupaniana*, 2n = 38; (**J**) *E. palustris*, 2n = 40; (**K**) *E. distans*, 2n = 40; (**L**) *Neottia cordata*, 2n = 38; (**M**) *N. ovata*, meiotic metaphase I showing 17 bivalents plus one B chromosome (arrowed); (**N**) *N. ovata*, metaphase chromosomes plus one B chromosome (arrowed), 2n = 34 + 1B; (**O**) *N. nidus-avis*, 2n = 36. Scale bar = 5 µm.

**Figure 3:**
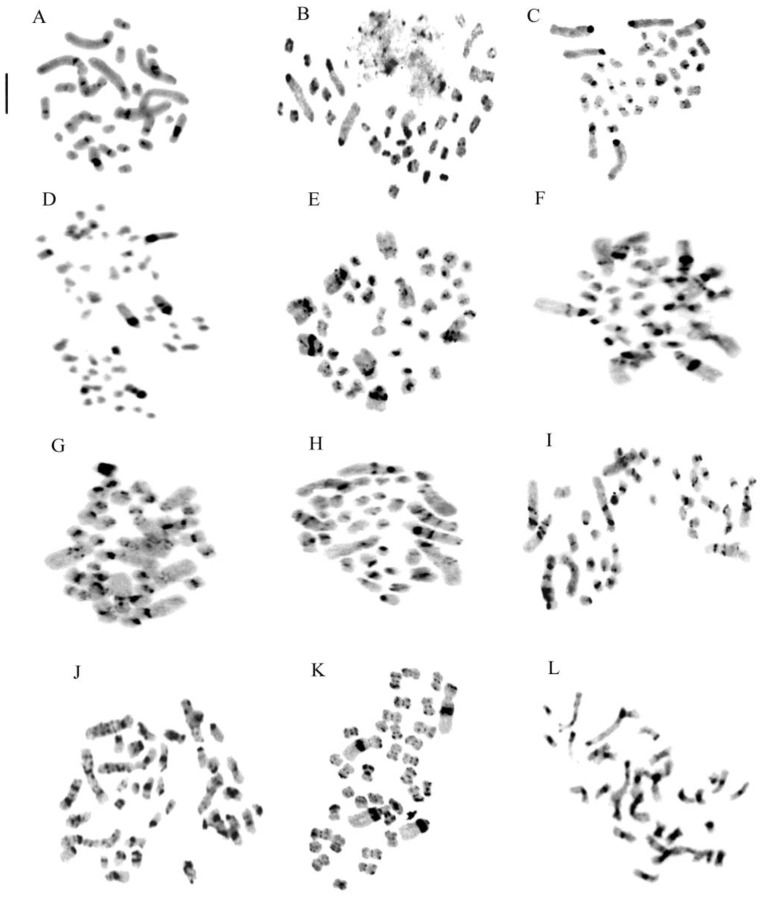
Somatic metaphases stained with the Giemsa banding technique in species of the Neottieae tribe. (**A**) *Cephalanthera longifolia*; (**B**) Sardinian *C. damasonium*; (**C**) Sardinian and continental Italy *C. damasonium*; (**D**) *Limodorum abortivum*; (**E**) *Epipactis helleborine*; (**F**) *E. placentina*; (**G**) *E. microphylla*; (**H**) *E. cupaniana*; (**I**) *E. distans*; (**J**) *E. palustris*; (K) *Neottia cordata*; (**L**) *N. nidus-avis*. Scale bar = 5 µm.

The staining methods used in this study include the Feulgen method for chromosomal counting and karyomorphological analysis, Giemsa band staining to detect constitutive heterochromatin (Figure 2 and Figure 3), and Hoechst 33258 and CMA3 fluorochrome staining to identify regions with repeated sequences rich in A-T and G-C.

### 2.1. Genus Cephalanthera Rich

**Figure 4:**
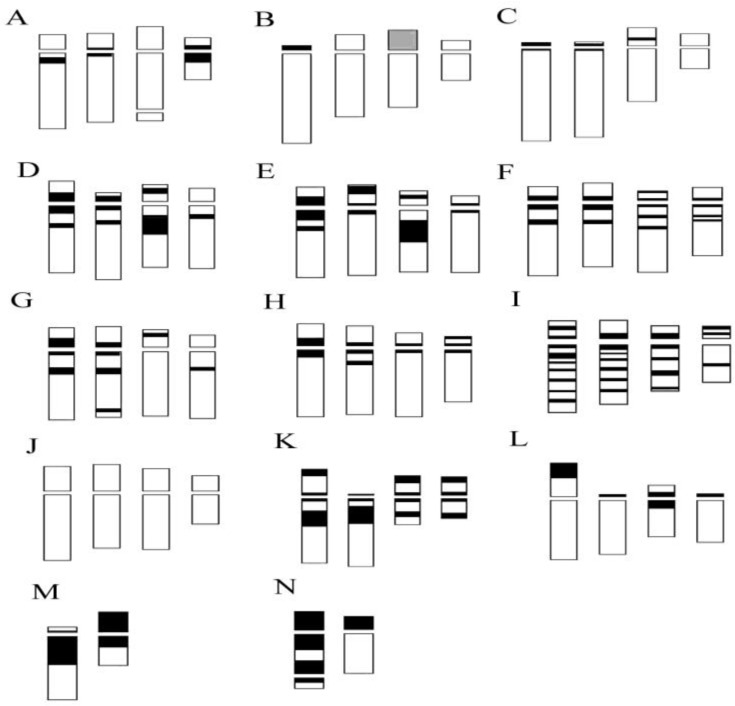
Partial ideograms (first four homologues) showing heterochromatin distribution in the longest chromosomes of (**A**) *Cephalanthera longifolia*; (**B**) *C. damasonium* Sardinia; (**C**) *C. damasonium* continental; (**D**) *Epipactis helleborine*; (**E**) *E. placentina*; (**F**) *E. distans*; (**G**) *E. cupaniana*; (**H**) *E. microphylla*; (**I**) *E. palustris*; (**J**) *Neottia ovata*; (**K**) *N. cordata*; (**L**) *N. nidus-avis*; (**M**) *Limodorum abortivum*; (**N**) *L. brulloi*.

**Figure 5:**
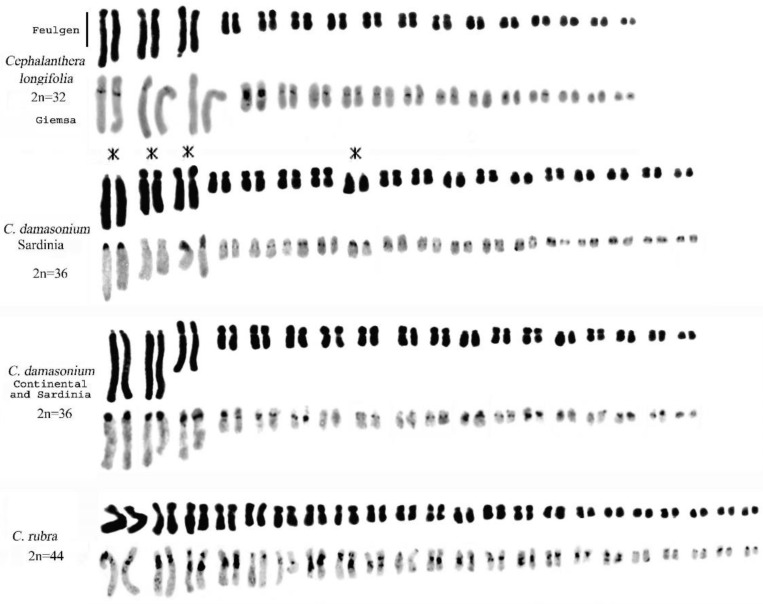
Feulgen-stained and Giemsa C-banded karyotypes of *Cephalanthera longifolia*, *C. damasonium*, and *C. rubra*. It is possible to trace the probable origin of the Continental *C. damasonium* karyotype from *C. longifolia* mediated by the cytotype found in a population of *C. damasonium* in Sardinia, via rearrangement in the first three pairs of long chromosomes and in one pair of small chromosomes (asterisks). Scale bar = 5 µm.

Our analyses confirm chromosome numbers 2n = 32 for *C. longifolia* (L.) Fritsch, 2n = 36 for *C. damasonium* (Mill.) Druce and 2n = 44 for *C. rubra* (L.) Rich., and they reveal the distribution of constitutive heterochromatin (Figure 2, Figure 3, Figure 4 and Figure 5). In *C. longifolia*, banding analyses with the Giemsa and Hoechst 33258 fluorochrome methods showed a subcentromeric band rich in A-T in pair 2. In *C. damasonium* banding analyses with the Giemsa and fluorochrome methods showed the short arm of pair 1 to be completely heterochromatic (Figure 3, Figure 4 and Figure 5), and the short arm of pair 2 to be partially heterochromatic with the telomeric region euchromatic. Pairs 3 and 5 showed a small heterochromatic band on the short arm close to the centromere. Giemsa banding in *C. rubra* showed numerous chromosomes rich in constitutive heterochromatin (Figure 5).

### 2.2. Genus Limodorum Boehm. in Ludwig

**Figure 6:**
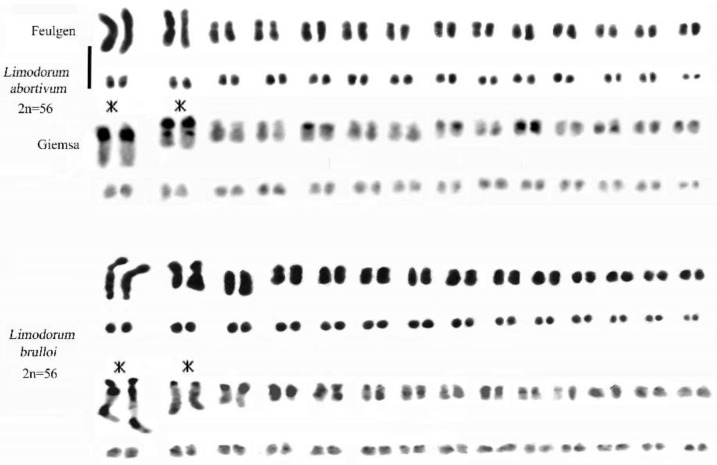
Feulgen-stained and Giemsa C-banded karyotypes of *Limodorum abortivum* (similar results were obtained for *L. trabutianum*) and *L. brulloi*. Note how the first two pairs of long chromosomes (with asterisks) show completely different karyomorphology and banding in the two species. Scale bar = 5 µm.

**Figure 7:**
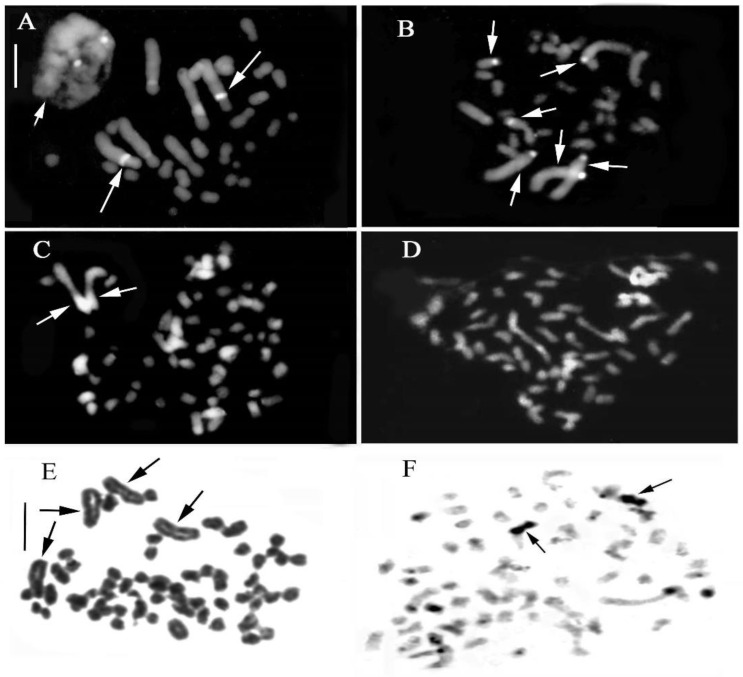
(**A**–**C**) Somatic metaphases stained with Hoechst 33258. (**A**) *Cephalanthera longifolia* 2n = 32; the short arrows indicate the interphase nucleus with two chromocentres, while the long arrows indicate subcentromeric bands. (**B**) *C. damasonium* 2n = 36; the arrows indicate heterochromatin bands. (**C**) *Limodorum abortivum* 2n = 56; the somatic metaphase stained with Hoechst 33258 arrows indicates heterochromatin bands. (**D**) *L. abortivum*; somatic metaphase stained with CMA3. (**E**) *L. trabutianum*, 2n = 60; somatic Feulgen-stained metaphases (arrows indicate long chromosomes) observed in specimens from various populations in Sardinia. (**F**) *L. trabutianum*; somatic metaphases stained with the Giemsa banding technique showed a broad band of constitutive heterochromatin only in the second pair of long chromosomes (arrows indicate chromosomes showing terminal bands). Scale bar = 5 µm.

Our analyses confirmed the similar karyomorphology of *L. abortivum* (L.) Sw. and *L. trabutianum* Batt., whereas *L. brulloi* Bartolo & Pulv. showed differences in karyomorphology and banding (Figure 4 and Figure 6). Hoechst 33258 staining showed blocks rich in A-T in the first pair and some small chromosomes (Figure 7C). In contrast, CMA staining showed intercalary bands in long chromosomes (Figure 7D). Analysis of some specimens of *L. trabutianum* from Sardinia showed a karyotype with different banding in the pairs of long chromosomes (Figure 7E,F). Indeed, in the first pair, the constitutive heterochromatin present in other specimens was not observed (Figure 7F).

### 2.3. Genus Epipactis Zinn

In this study we confirm the chromosome numbers 2n = 2x = 38 and 2n = 2x = 40 for all species of *Epipactis* examined. 

**Figure 8:**
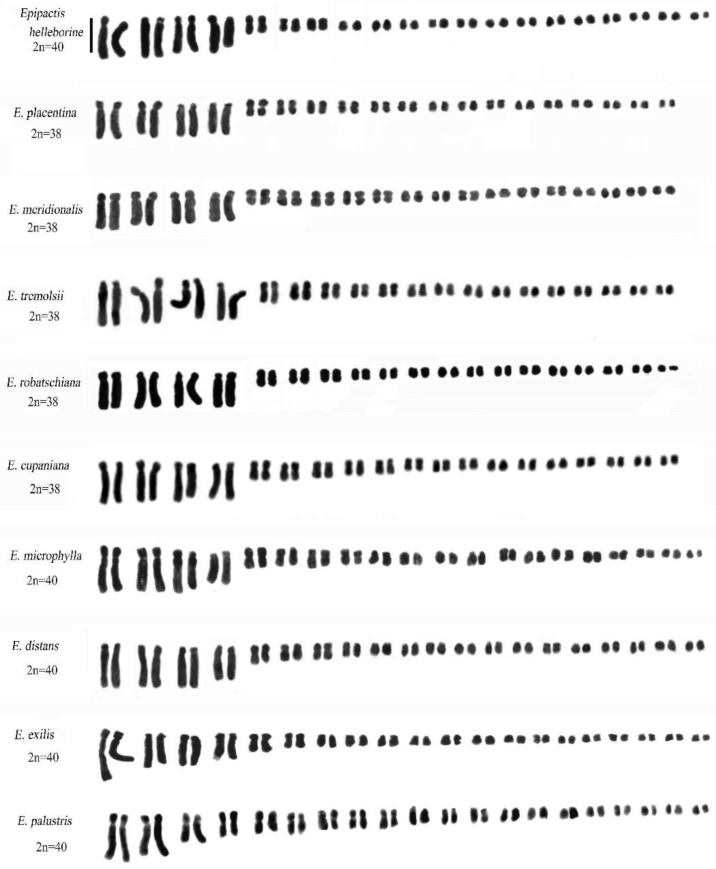
Feulgen karyotypes in *Epipactis* species. Scale bar = 5 µm.

Some species, such as *Epipactis helleborine* (L.) Crantz, *E. placentina* Bongiorni & Grünanger, *E. tremolsii* Pau, *E. muelleri* Godfery, *E. meridionalis* H. Baumann & R. Lorenz, *E. schubertiorum* Bartolo, Pulv. & Robatsch, *E. robatschiana* Bartolo, D’Emerico, Pulv., Terrasi & Stuto, and *E. distans* Arv.-Touv., show similar karyotypes, although *E. helleborine* and *E. distans* differ from *E. placentina*, *E. tremolsii*, and *E. muelleri* (2n = 38) in their chromosome number 2n = 40 (Figure 8). 

**Figure 9:**
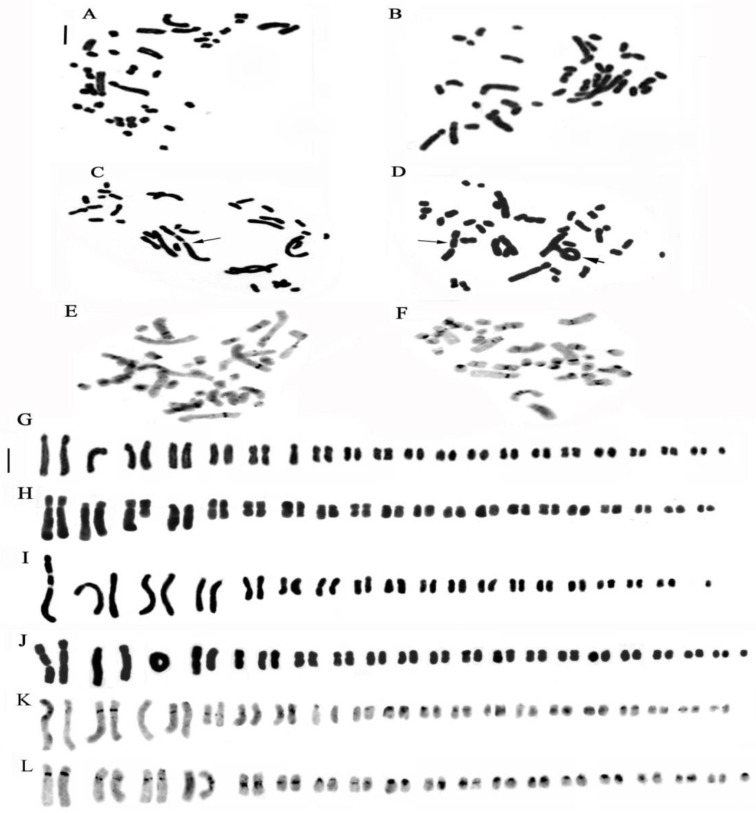
(**A**–**F**) Structural alterations in *Epipactis microphylla*. Numerous somatic metaphases exhibited aneuploidy and evident structural alterations in some chromosomes. (**A**,**B**) Somatic metaphases with 38 (**A**) and 39 (**B**) chromosomes. (**C**) Somatic metaphase with 36 chromosomes; the long arrow indicates rearrangement. (**D**) Somatic metaphase with 39 chromosomes; the short arrow indicates a ring chromosome and the long arrow indicates chromosome rearrangement. (**E**,**F**) Somatic metaphases stained with the Giemsa banding technique. (**G**–**L**) Karyotypes following Feulgen and Giemsa staining ((**I**) karyotype of (**C**), (**J**) karyotype of (**D**), (**K**) karyotype of (**E**) and (**L**) karyotype of (**F**)). (**H**) Somatic metaphase with 40 chromosomes; note the evident heteromorphy in the second and third pairs. (**K**) In the first chromosome of the karyotype, it is possible to notice a double band of constitutive heterochromatin, probably linked to the first chromosome observed in (**I**). Scale bar = 5 µm.

One specimen of *E. microphylla* (Ehrh.) Sw. showed aneuploidy with chromosome numbers 36, 37, 38, and 39. Some metaphase plates showed evident alteration of the karyotype due to the presence of mutations in some long chromosomes (Figure 9C,D).

**Figure 10:**
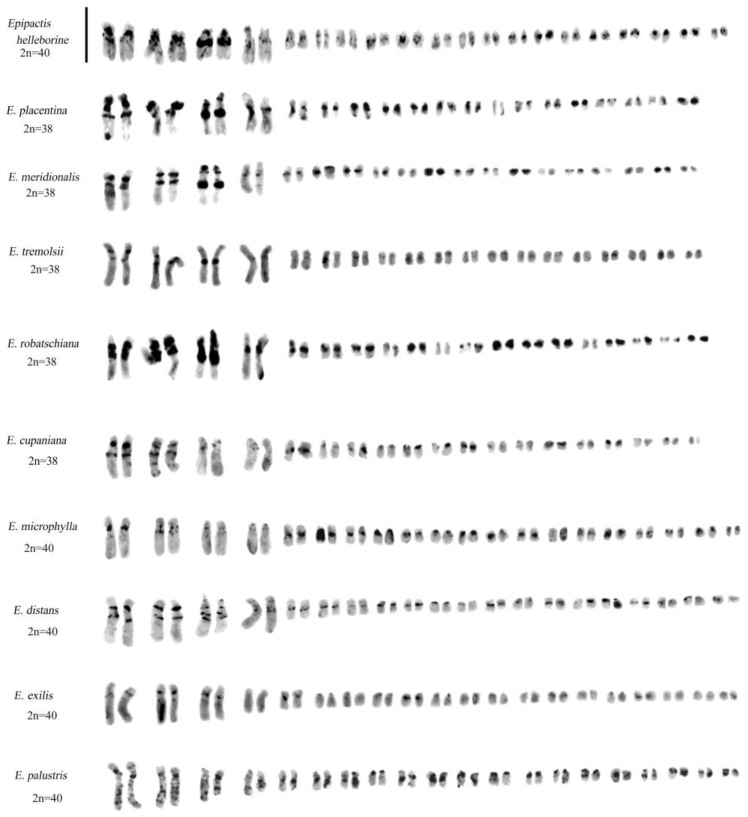
Giemsa C-banded karyotypes in *Epipactis* species. Note that the species *E. helleborine*, *E. placentina*, *E. meridionalis*, *E. tremolsii*, and *E. robatschiana* show a characteristic broad band in an intercalated position on the long arm of chromosome pair 3. The absence of this heterochromatic band in *E. cupaniana*, *E. distans*, *E. exilis*, and *E. microphylla* is the main feature that differentiates them from species of the *E. helleborine* group. Scale bar = 10 µm.

Investigation with the Giemsa banding technique confirmed the presence of heterochromatin, mainly in the long chromosomes (Figure 4 and Figure 10). Indeed, in *Epipactis helleborine*, *E. placentina*, *E. tremolsii*, *E. muelleri*, *E. meridionalis*, *E. schubertiorum*, and *E. robatschiana*, Giemsa banding confirmed the presence in pair 3 of a large band on the long arm near the centromere. In contrast, *E. aspromontana* Bartolo, Pulv. & Robatsch, *E. microphylla*, *E. cupaniana* C. Brullo, D’Emerico & Pulv., *E. exilis* P. Delforge, *E. distans*, and *E. palustris* (L.) Crantz showed different heterochromatin patterns in the first four pairs of chromosomes (Figure 8 and Figure 10).

### 2.4. Genus Neottia Guett. (Including Listera)

**Figure 11:**
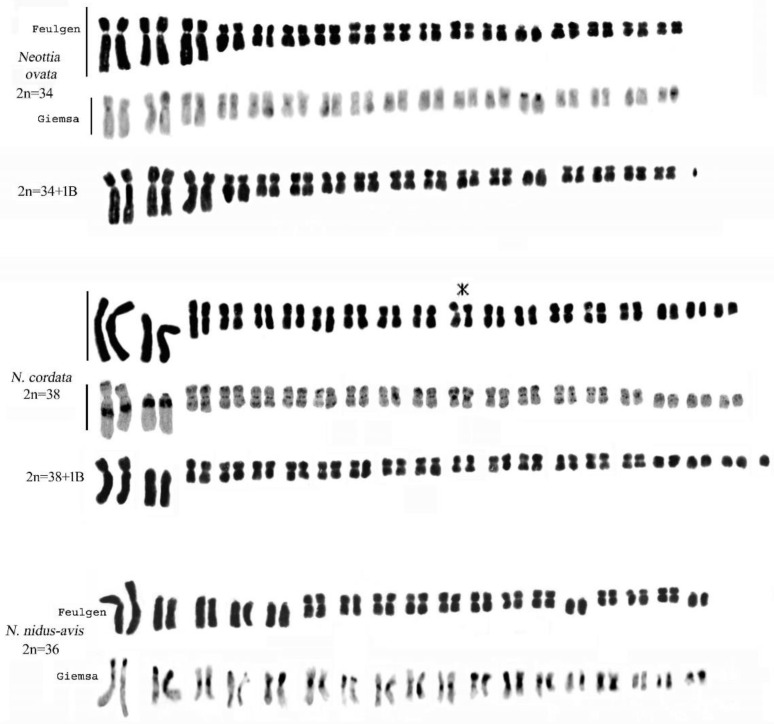
Feulgen-stained and Giemsa C-banded karyotypes of *Neottia ovata*, *N. cordata*, and *N. nidus-avis*. Both *N. ovata* and *N. cordata* showed specimens with one B-chromosome. In *N. cordata*, it is possible to observe a secondary constriction near the centromere in pair 11 (asterisk). Scale bar = 10 µm.

Our *Neottia* observations confirmed 2n = 34 chromosomes for *N. ovata* (L.) Hartm., 2n = 38 for *N. cordata* (L.) Rich., and 2n = 36 for *N. nidus-avis* (L.) Rich. However, in some specimens of *N. ovata* and *N. cordata*, 2n = 34 + 1B and 2n = 38 + 1B were observed, respectively (Figure 2 and Figure 11). In *N. nidus-avis*, numerous chromosomes with terminal bands rich in constitutive heterochromatin were highlighted with Giemsa banding (Figure 11).

### 2.5. Plot of the Morphometric Parameters Mca (Mean Centromeric Asymmetry) and CVcl (Coefficient of Variation of Chromosome Length)

**Figure 12:**
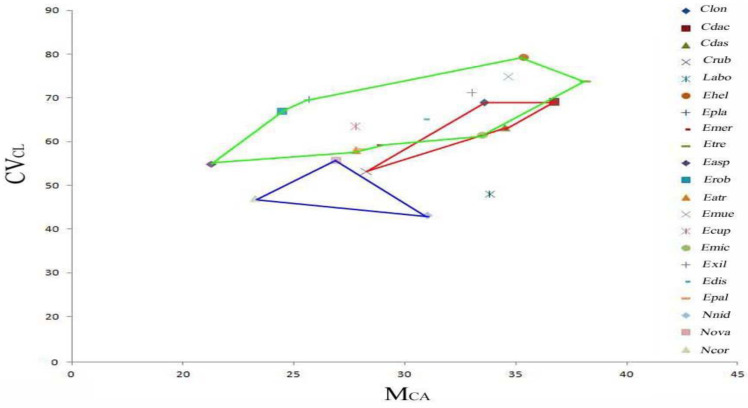
Mca and CVcl values of the analysed species’ karyotypes. The green polygon represents *Epipactis* taxa, the red polygon *Cephalanthera*, the blue polygon *Neottia*, and the blue asterisk *Limodorum*.

We used the asymmetry indices Mca and CVcl to draw the plot depicted in Figure 12 showing the relative position of all species considered in this study.

It is evident from the plot that the four genera share a similar pattern of asymmetry, although some *Epipactis* and *Cephalanthera* species have a higher Mca value than *Neottia* species. Furthermore, it is interesting to note the asymmetry indices of *Cephalanthera longifolia* and continental and Sardinian *C. damasonium*, which reflect the cytomorphology of these entities.

## 3. Discussion 

### 3.1. Cytotaxonomy between Groups and Species

Four genera of the *Neottieae* tribe are found in the Mediterranean region: *Cephalanthera*, *Epipactis*, *Limodorum*, and *Neottia* (including *Listera*) [4]. 

The cytological examination of this tribe revealed variability in the number of chromosomes. Across all studied genera, there was confirmation of an asymmetric “standard” karyotype classified as “bimodal”, wherein chromosome lengths displayed a distinct distribution, forming two or three groups of varying sizes [31]. This bimodal characteristic aligns with the typical karyotypic pattern reported in the literature for the Neottieae tribe.

The karyological examination of various taxa within the Neottieae tribe yielded interesting insights into their phylogenetic relationships. A comparative analysis of their karyotypes indicates a degree of similarity, further corroborated by the distribution of constitutive heterochromatin [24]. Moreover, the Giemsa banding technique revealed that the examined species exhibit varying distributions of heterochromatin, predominantly located on the larger chromosomes [24,26,49].

Dressler [3] proposes the genus *Cephalanthera* as the foundational group within the Neottieae tribe, from which the *Listera* group is derived, supported by molecular data [50]. Karyological analyses of the genus *Cephalanthera* have shown a wide range of chromosomal numbers with 2n = 32, 34, 36, 42, 44, 48, 64, and 68 [51]. 

*Cephalanthera damasonium* is a species in which autogamy and cleistogamy can occur. The species consistently exhibited 2n = 36 chromosomes across all examined populations. Interestingly, within this species, the number, morphology, and banding pattern of metaphase chromosomes remained similar across all *C. damasonium* specimens studied in Europe, with minor structural variations, suggesting karyotype stability [24,52]. 

Regarding the species *C. longifolia*, several hypotheses concerning its ancestral origin within the *Cephalanthera* group can be proposed. Notably, it has a broader geographical distribution than all other species in the group [2]. Furthermore, both the chromosome number (2n = 32) and its karyotype appear to present cytogenetic stability [17,20,24,27,30,52,53,54]. In addition, karyotypes resembling that of *C. longifolia* have been observed in *C. erecta* Blume and *C. falcata*, both with a chromosome number of 2n = 34 and of Asian origin. Interestingly, the first three pairs of chromosomes in these species are identical to those observed in *C. longifolia*, as noted by Yang & Zhu [53]. Furthermore, phylogenetic analyses within the Neottieae tribe using molecular data indicate an evolutionary origin of *C. erecta* and *C. falcata* from *C. longifolia* [5]. Based on these findings, it is possible to suggest *C. longifolia* as an ancestor species in the genus *Cephalanthera*.

The genus *Limodorum* includes entities characterised by a high chromosomal number (2n = 54, 56, 58, 60) [24,30,55]. This group is represented by three morphologically distinct species: *L. abortivum* characterised by two stem cells, *L. trabutianum* by three stem cells, and *L. brulloi* by five stem cells [56]. The three entities within the genus *Limodorum* exhibit certain relationships with species from the genera *Cephalanthera* and *Epipactis* due to their possession of bimodal karyotypes [24,25].

A comprehensive karyological analysis of the three *Limodorum* species unveiled differences in the first two pairs of long chromosomes. In both *L. abortivum* and *L. trabutianum*, the first pair of chromosomes appears telocentric, featuring a prominent heterochromatic band on the long arm near the centromere. The second pair exhibits a significant band around the secondary constriction on the short arm. On the other hand, in a population of *L. trabutianum* from Sardinia, banding with Giemsa showed the presence of constitutive heterochromatin only in the second pair. Conversely, the karyotype and chromosome banding of *L. brulloi* reveal a distinct structure and quantity of constitutive heterochromatin, clearly distinguishing it from the other two species within the genus. 

The genus *Epipactis* includes more than thirty entities divided into two sections: *Arthrochilum* and *Epipactis* [11]. Bearing in mind the classification reported by GIROS [7], in our study, we considered species representative of both.

A range of chromosomal numbers are reported in the literature, with the most frequently cited being 2n = 38 and 2n = 40. All species possess a bimodal karyotype consisting of four large and fourteen/fifteen small chromosome pairs [17,23,24,26,28,49]. Based on the cytological analyses presented here, the karyomorphological traits and the distribution of constitutive heterochromatin highlight a slightly different constitution of genomes within the *E. helleborine* group. Moreover, our analyses conducted using the Giemsa banding technique showed one or two pairs of small chromosomes in all examined species, characterised by a fully heterochromatic short arm. These formations probably originated from the centromeric fission of certain metacentric or submetacentric chromosomes, followed by the amplification of repeated DNA sequences [57].

In the *Epipactis* group, *E. cupaniana*, *E. distans*, *E. exilis*, and *E. microphylla* show similarities in karyomorphology and heterochromatic pattern. The absence of heterochromatic bands on the largest chromosomal pairs is the main feature that differentiates them from species of the *E. helleborine* group [24,49]. Morphological, karyological, and phytogeographical studies of the known taxa of the *Epipactis helleborine* group have revealed a distinct differentiation within the Sicilian population. For instance, the species *E. cupaniana*, like other species within this genus, exhibits a diploid chromosome set of 2n = 38. The karyotype is asymmetric and consists of 8 large and 30 small chromosomes. Moreover, with the Giemsa banding technique, pairs 1 and 2 show a medium-large centromeric band and an intercalary band on the long arm [49].

Another interesting species belonging to this group is *Epipactis aspromontana*. Morphological investigations have shown that *E. aspromontana* shows affinity with both *E. leptochila* (Godfery) Godfery and *E. helleborine* s.l. [58]. The chromosomal number of *E. aspromontana* is diploid, with 2n = 38. However, karyomorphological analyses show no close affinity between *E. aspromontana* and the taxa belonging to the *E. helleborine* group. Indeed, in the latter, numerous species are characterised by the presence in the third pair of a wide band on the long arm, absent in *E. aspromontana*. Conversely, a characteristic of *E. aspromontana* is the presence of an evident large heterochromatic band that occupies almost the entire short arm of the second pair of long chromosomes. Moreover, our observations identified a polyploid specimen of this species, in which metaphase I plates of meiosis showed univalent, bivalent, and trivalent figures. The specimen also yielded an aneuploid series with the chromosomal number ranging between 50 and 53. 

In terms of its chromosomal structure and heterochromatin distribution, *E. palustris*, belonging to the section *Arthrochilum* Irmisch, shows a clear separation from other species of the genus. Indeed, molecular investigations confirm its separation from species belonging to the *Epipactis* section [4,8,59]. In addition, *E. palustris* shows chromosome number 2n = 40. The karyotype includes two pairs of large chromosomes (1 and 2), one pair of medium-large chromosomes (3), and the remainder of decreasing length. Using the Giemsa banding method, the chromosomes showed remarkable band diversity.

Regarding the *Epipactis* group, on the basis of molecular analyses, Tranchida-Lombardo et al. [60] advance the hypothesis of recent colonisation by the *Epipactis* group of the Italian peninsula, which, thus, acted as a centre of diversification.

Another aspect of the *Epipactis* genus is the variability in chromosome numbers reported by numerous researchers. As previously mentioned, the most frequently observed chromosome numbers in many species are 2n = 38 and 40. However, a range of other chromosome numbers have been documented for the genus *Epipactis*, including 2n = 18, 24, 32, 34, 36, 44, 46, and 48 [4,23]. Furthermore, as with other genera within the Neottieae tribe, species within the *Epipactis* group exhibit a bimodal karyotype characterised by the presence of two sets of chromosomes of contrasting sizes. These sets originate from different ancestral processes, as observed in genera such as *Cephalanthera* [27,37,61].

The genus *Neottia* includes a group of species with variable chromosomal numbers and a base number x = 17, 18. In the Mediterranean region the genus is represented by the species *N. cordata* (2n = 38), *N. nidus-avis* (2n = 36), and *N. ovata* (2n = 34). 

In this study, conventional chromosome staining showed that the karyological features of *N. cordata* and *N. ovata* generally exhibit similar chromosomal structures. However, the karyotype of *N. cordata* differs from that of *N. ovata* in having four pairs of telocentric chromosomes [25]. Heterochromatic bands in *N. ovata* and *N. cordata* showed considerable differences in the amount and distribution of heterochromatin. In *N. ovata*, there are modest centromeric bands and the presence of heterochromatin is limited to chromosome pair 9, while *N. cordata* chromosomes show a high number of constitutive heterochromatin bands.

*Neottia nidus-avis*, with the chromosomal number 2n = 36, is an interesting entity, with a karyotype made up of numerous telocentric chromosomes. Its karyomorphology is very interesting in that it accords with the chromosomal alteration responsible for karyotypic evolution [28]. 

### 3.2. Chromosome Alterations and Heterochromatin Distribution

Chromosomal mutations have been suggested by some authors as being the origin of the karyotype in the genus *Cephalanthera* [25,27,52,62], via Robertsonian rearrangement events leading to karyotype differentiation between *C. longifolia* and *C. damasonium*. An interesting case was reported by Yang and Zhu [53] in *C. falcata* Blume from China. In this species, two populations were identified with the same chromosome number (2n = 34), but with a difference in the karyotype. Indeed, in one of the populations, the first and third pairs were affected by a rearrangement of the chromosomes via translocation. A similar result was observed in two Sardinian populations of *C. damasonium* [25], with a comparison of karyological data indicating that the two populations have different cytotypes but the same chromosomal number. These variations imply an alternative hypothesis regarding the evolution of the *C. damasonium* karyotype [27]: the chromosomal number 2n = 36 might have evolved from an ancestral species with a chromosomal number of 2n = 32, probably derived from *C. longifolia*, via rearrangements of the karyotype caused by processes such as fission, inversion, and translocation. The karyomorphology and distribution of heterochromatin suggest that the karyotype of *C. damasonium* found in a population in Sardinia may be more ancestral than those found in continental areas. The genetic differentiation into two distinct cytotypes suggests that geographical barriers played a role in the initial isolation of a new cytotype from the ancestral one. Interestingly, despite the differences in karyotypes, there was no significant impact on plant morphology [25].

In the *Limodorum* genus, *L. brulloi* differs from *L. abortivum* and *L. trabutianum* in having the short arm of the first subtelocentric pair entirely heterochromatic and in having a subcentromeric band close to the centromere. The karyological data indicate that *L. abortivum* and *L. trabutianum* underwent greater chromosomal rearrangement during evolution than the *L. brulloi* species. Furthermore, the karyological differences observed in the three species confirm morphological studies that characterise *L. brulloi* as ancestral with respect to *L. abortivum* and *L. trabutianum* [55].

In the *Epipactis* group, it is noteworthy that several species with a chromosome number of 2n = 38, including *E. muelleri*, *E. placentina*, *E. meridionalis*, *E. tremolsii*, *E. schubertiorum*, and *E. robatschiana*, exhibit a distinctive broad band in an intercalary position on the long arm of chromosome pair 3, resembling what is found in *E. helleborine*. Due to this similarity, many of these species have been synonymously classified as *E. helleborine* [2]. It appears probable that the species constitute a genetically cohesive group, wherein each taxon might represent a morphotype of the same *E. helleborine* species, as noted by Rewicz et al. [63] and Sramkò et al. [8]. Moreover, it is plausible to suggest that these species potentially originated from epigenetic phenomena, with *E. helleborine* serving as the likely ancestral species. Epigenetics primarily focuses on the investigation of inheritable alterations in phenotype that occur without changes to DNA sequences [64,65]. Indeed, various environmental factors may have influenced DNA methylation in the third pair of long chromosomes. Further exploration via molecular and biochemical analyses will be crucial in elucidating our cytogenetic investigations and potentially offering insights into the taxonomic challenges within the genus *Epipactis*. 

Bimodal karyotypes, with their distinctive complements, may be more susceptible to chromosomal rearrangement [32,40,43]. In this context, an interesting case arose in our study with the discovery of a genome anomaly in a specimen of *E. microphylla*. Karyological analyses revealed aneuploidy in the chromosome count, which ranged from 36 to 40. Moreover, certain somatic metaphases exhibited evident structural alterations in some chromosomes, indicative of rearrangements resulting from fission and translocation, and in this specimen, a ring chromosome was also observed [66,67,68]. This chromosome usually results from the union of a broken end of a chromosome with the opposite telomeric region and may occur spontaneously [46]. Based on this find, it is possible that, in many cases, the different chromosome counts are due to the high incidence of chromosomal rearrangement, which can easily occur in the bimodal chromosome set in natural populations [40,69,70]. 

With a higher telomeric heterochromatin content, *N. cordata* would appear to be subject to profound restructuring of the karyotype. Previous studies have identified heterochromatin content as an indicator of evolution in other plants [71,72]. Therefore, it is assumed that the differentiation of the previous karyotype is the result of structural rearrangements of the chromosomes, possibly the result of centric fission in the second long pair and a medium-sized pair.

In *N. nidus-avis*, heterochromatic bands were observed in centromeric and telomeric positions. This species exhibits an asymmetric karyotype, centromeric heterochromatin, and numerous chromosomes with a heterochromatic short arm. Telocentric chromosomes may have originated from the centric fission of metacentric chromosomes with the subsequent amplification of heterochromatin [57,73].

## 4. Materials and Methods

### 4.1. Cytological Analysis

The examined taxa and their collection sites are shown in Table 1. Mitotic chromosomes were observed in the tissues of immature ovaries. At least ten metaphases were examined, and the karyotype was constructed from well-spread metaphase plates. Immature ovary tissues were pre-treated with 0.3% colchicine at room temperature for 2 h. For Feulgen staining, they were fixed in 3:1 (*v*/*v*) ethanol–glacial acetic acid and stored in the deep-freeze for up to several months. Hydrolysis was performed at 20 °C in 5.5 N HCl for 20 min [74]. The material was then stained in freshly prepared Feulgen stain. 

For C-banding, immature ovaries were fixed in 3:1 (*v*/*v*) ethanol–glacial acetic acid and stored in the deep-freeze for up to several months. Subsequently, they were squashed in 45% acetic acid. Coverslips were removed by the dry ice method and the preparations air-dried overnight. The slides were then immersed in 0.2 N HCl at 60 °C for 3 min, thoroughly rinsed in distilled water, and then treated with 4% Ba(OH)2 at 20 °C for 4 min. After thorough rinsing, they were incubated in 2 × SSC at 60 °C for 1 h. The stain used was 3–4% Giemsa (BDH) at pH 7.

For Hoechst 33258 staining, squash preparations were made up as they were for C-banding and were then stained in a 2 µg/mL dye solution in pH 7 McIlvaine buffer for 5 min, rinsed, and mounted in 1:1 *v*/*v* buffer–glycerol [75].

For chromomycin A3 (CMA) staining, slides were stained with 0.5 mg/mL CMA for 1 h and mounted in 1:1 (*v*/*v*) pH 7.0 McIlvaine buffer–glycerol.

### 4.2. Chromosome Numbers and Karyotype Parameters

Chromosome pairs were identified and arranged based on length. The nomenclature used for describing karyotype composition follows Levan et al. [76], who denote centromeric positions using the terms “median (arm ratio 1.0–1.7)”, “submedian (a.r. 1.7–3.0)”, “subterminal (a.r. 3.0–7.0)”, and “terminal (a.r. 7.0–∞)”. Karyotype morphometric characters were evaluated by calculating the haploid complement, while the karyotype asymmetry indices M_CA_ (Mean Centromeric Asymmetry) and CV_CL_ (Coefficient of Variation of Chromosome Length) were used for the evaluation of karyotype asymmetry. CV_CI_ (Coefficient of Variation of the Centromeric Index) was used to evaluate heterogeneity in the position of the centromeres [77,78,79].

Chromosome measurements were conducted using the freeware IdeoKar 1.2 (http://agri.uok.ac.ir/ideokar/index.html, accessed on 10 September 2023). The plot of the karyotype M_CA_ and CV_CL_ values was generated using the freeware Open Office 4.1.14 program.

### 4.3. Nomenclature

Regarding the nomenclature of species, we followed GIROS [7] and, in some cases, POWO [2].

## 5. Conclusions

The Neottieae tribe serves as an excellent model for testing rearrangement hypotheses in chromosomes. In this study, we observed chromosomal alterations of significant interest in many species of this tribe. Indeed, within the genera *Cephalanthera*, *Epipactis*, *Limodorum*, and *Neottia*, important cases have been described where rearrangement of the chromosomal set of ancestral entities may have contributed to the formation of current species.

One of the most interesting cases lies within the genus *Epipactis*, where, from a cytogenetic perspective, *E. helleborine* seems to serve as the direct ancestor of many species within the genus. This group is currently undergoing evolutionary radiation, exhibiting a wide array of genotypes, phenotypes, and responses to environmental factors. Given these fascinating findings and the complexity of morphological, cytogenetic, and molecular issues within *Epipactis* species, it is plausible to suggest that epigenetic processes play a role in numerous entities within the group.

Finally, from the results obtained, it is possible to note that the karyomorphological variations, both structural and in the distribution of constitutive heterochromatin, suggest that these parameters seem to play an important role in the evolution of the karyotype in many entities belonging to the Neottieae tribe.

## Figures and Tables

**Table 1:** Taxon, code, provenance, chromosome number, formula, and morphometric parameters (average values) in *Cephalanthera*, *Limodorum*, *Epipactis*, and *Neottia* species. THL = total chromosome length of the haploid complement; M_CA_ = Mean Centromeric Asymmetry; CV_CL_ = Coefficient of Variation of Chromosome Length; CV_CI_ = Coefficient of Variation of Centromeric Index. Chromosome abbreviations: *m*, metacentric; *sm*, submetacentric; *st*, subtelocentric; *t*, telocentric.

Taxon	Code	Provenance	2n	Formula	THL	M_CA_	CV_CL_	CV_CI_
*Cephalanthera damasonium* (Mill.) Druce	Cdac	Continental Italy, Sardinia	36	12 *m* + 16 *sm* + 4 *st* + 4 *t*	68.96	36.73	69.08	36.08
*C. damasonium* (Mill.) Druce	Cdas	Sardinia	36	16 *m* + 12 *sm* + 4 *st* + 4 *t*	51.88	34.52	63.17	38.64
*C. longifolia* (L.) Fritsch	Clon	Italy	32	10 *m* + 16 *sm* + 6 *st*	60.00	33.57	68.86	25.40
*C. rubra* (L.) Rich.	Crub	Italy	44	22 *m* + 16 *sm* + 6 *st*	66.28	28.26	53.09	24.64
*Limodorum abortivum* (L.) Sw.	Labo	Italy	56	18 *m* + 26 *sm* + 12 *st*	59.57	33.83	47.98	26.46
*Epipactis aspromontana* Bartolo, Pulv. & Robatsch	Easp	Italy	38	30 *m* + 4 *sm* + 4 *st*	50.67	21.30	54.82	22.71
*E. atrorubens* (Hoffm.) Besser	Eatr	Italy	38	20 *m* + 10 *sm* + 8 *st*	48.70	27.80	57.97	30.29
*E. cupaniana* C. Brullo, D’Emerico & Pulv.	Ecup	Italy	38	24 *m* + 4 *sm* + 10 *st*	52.77	27.79	63.50	30.37
*E. distans* Arv.-Touv.	Edis	Italy, France	40	20 *m* + 10 *sm* + 10 *st*	52.88	30.90	65.09	30.66
*E. exilis* P. Delforge	Exil	Italy	40	16 *m* + 18 *sm* + 6 *st*	57.15	33.02	71.20	26.48
*E. helleborine* (L.) Crantz	Ehel	Italy	40	16 *m* + 14 *sm* + 10 *st*	50.90	35.35	79.28	29.90
*E. meridionalis* H. Baumann & R. Lorenz	Emer	Italy	38	20 *m* + 8 *sm* + 10 *st*	44.76	28.78	59.21	30.12
*E. microphylla* (Ehrh.) Sw.	Emic	Italy	40	16 *m* + 12 *sm* + 12 *st*	63.02	33.50	61.46	34.63
*E. muelleri* Godfery	Emue	Italy	38	16 *m* + 12 *sm* + 10 *st*	47.43	34.67	74.89	28.56
*E. placentina* Bongiorni & Grünanger	Epla	Italy	38	26 *m* + 4 *sm* + 8 *st*	45.25	25.71	69.64	27.72
*E. palustris* (L.) Crantz	Epal	Italy	40	24 *m* + 8 *sm* + 8 *st*	63.60	28.05	58.28	24.36
*E. robatschiana* Bartolo, D’Emerico, Pulv., Terrasi & Stuto	Erob	Italy	38	26 *m* + 4 *sm* + 8 *st*	51.08	24.48	66.98	29.13
*E. tremolsii* Pau	Etre	Italy	38	16 *m* + 8 *sm* + 10 *st* + 4 *t*	52.83	38.14	73.74	38.99
*Neottia cordata* (L.) Rich. (*Listera*)	Ncor	Italy	38	28 *m* + 4 *sm* + 4 *st* + 2 *t*	38.61	23.28	47.04	36.06
*N. nidus-avis* (L.) Rich.	Nnid	Italy	36	24 *m* + 4 *sm* + 2 *st* + 6 *t*	66.26	31.03	43.07	42.53
*N. ovata* (L.) Hartm. (*Listera*)	Nova	Italy	34	22 *m* + 10 *sm* + 2 *st*	58.59	26.92	55.69	22.00

## Data Availability

Data are contained in the article.
